# Predicting insulin resistance using the triglyceride-to-high-density lipoprotein cholesterol ratio in Taiwanese adults

**DOI:** 10.1186/1475-2840-10-93

**Published:** 2011-10-17

**Authors:** Jui-Kun Chiang, Ning-Sheng Lai, Jiunn-Kae Chang, Malcolm Koo

**Affiliations:** 1 Department of Family Medicine, Buddhist Dalin Tzu Chi General Hospital, Chiayi, Taiwan; 2 Department of Biotechnology, Chia Nan University of Pharmacy & Science, Tainan, Taiwan; 3 Department of Allergy, Immunology and Rheumatology, Buddhist Dalin Tzu Chi General Hospital, Chiayi, Taiwan; 4 School of Medicine, Tzu Chi University, Hualien, Taiwan; 5 Dalla Lana School of Public Health, University of Toronto, Ontario, Canada

**Keywords:** prediction, insulin resistance, Chinese, ethnic groups

## Abstract

**Background:**

The triglyceride to high-density lipoprotein cholesterol ratio (TG/HDL-C) has been advocated as a simple clinical indicator of insulin resistance. Thresholds of TG/HDL-C appeared to depend on ethnicity. However, no studies have specifically compared the accuracy of TG/HDL-C with and without other clinical and demographic factors in predicting insulin resistance in Taiwanese adults. The aim of the present investigation was to use TG/HDL-C and other clinical available factors to predict insulin resistance in Taiwanese adults.

**Methods:**

A total of 812 subjects were recruited from at the time of their general health examination at the Buddhist Dalin Tzu Chi General Hospital, Taiwan. Demographic information and clinical characteristics were obtained. Insulin resistance was defined by the homeostasis model assessment for insulin resistance (HOMA-IR). Simple and multiple logistic regression analyses were used to obtain probabilities of insulin resistance (HOMA-IR > 2) using TG/HDL-C with (Model 2) and without (Model 1) other clinical variables. A receiver operating characteristic (ROC) analysis was conducted to evaluate the ability of the two models to correctly discriminate between subjects of low and elevated HOMA-IR.

**Results:**

Female sex, greater waist circumferences, and higher ALT levels were significantly associated with the risk of elevated HOMA-IR in addition to TG/HDL-C in the multiple logistic regression (Model 2). The area under the ROC curve (AUC) of Model 2 was 0.71 [95% CI = 0.67-0.75] and was significantly higher (*P *= 0.007) than the AUC 0.66 [95% CI = 0.62-0.71] of Model 1.

**Conclusions:**

The diagnostic accuracy of insulin resistance, defined by HOMA-IR, using TG/HDL-C can be significantly enhanced by including three additional clinically available factors - sex, waist circumferences, and ALT levels.

## Background

Metabolic syndrome is characteristically defined as a clustering condition of cardiovascular risk factors including hyperglycemia, dyslipidemia, hypertension, and central obesity [1]. Its occurrence is strongly associated with increased risk in the development of diabetes and cardiovascular disease. A recent meta-analysis of 87 prospective observational studies reported that metabolic syndrome was associated with a two-fold increase in cardiovascular disease outcomes and a 50% increase in risk of all-cause mortality [2].

The pathophysiology of the metabolic syndrome remains a subject of controversy but many of its features are associated with insulin resistance. It is typically defined as decreased sensitivity or responsiveness to metabolic actions of insulin. Currently, there are several directly and indirect methods to assess insulin resistance [3]. The gold standard method was the hyperinsulinemic-euglycemic clamp (glucose clamp) technique, originally developed by DeFronzo [4]. However, the method is complex and time consuming, making it not feasible for either epidemiological investigations or routine clinical applications. Therefore, a number of surrogate indices of insulin resistance have been developed. Previous studies have shown that insulin resistance scores based on the homeostasis model assessment (HOMA) method was strongly correlated with glucose clamp-assessed insulin resistance [5,6]. The model utilize a set of empirically derived nonlinear equations to predict the homeostatic concentrations of fasting insulin and glucose, which reflect the varying degrees of pancreatic β-cell function and insulin resistance [7].

Although HOMR-IR has been widely used in the study of metabolic syndrome, the lack of standardized insulin assay has limited its clinical utility [8]. It would be of considerable benefit for clinicians if other standardized measures are available for predicting insulin resistance without the need for direct measurements of fasting insulin. Since hypertriglyceridemia and low high-density lipoprotein cholesterol are two key metabolic abnormalities associated with insulin resistance states [9,10], the triglyceride to high-density lipoprotein cholesterol ratio (TG/HDL-C) has been advocated as a simple clinical indicator of insulin resistance [11,12]. It has been evaluated as predictor of diabetes [13] and coronary heart disease [14]. In a cohort study on 3,014 patients, the area under the receiver operating characteristic curves (AUC) for predicting insulin resistance with TG/HDL-C was 0.75 [14]. Nevertheless, recent studies have reported that racial differences existed in the predictability of the ratio [15]. In a cross-sectional study of 50 white and 99 African American adults, the AUCs of TG/HDL-C for predicting insulin resistance, as measured by HOMA-IR, was found to be 0.77 and 0.64, respectively. It was concluded that the relationship between TG/HDL-C and insulin resistance varied in different ethnic groups and TG/HDL-C was not a reliable marker for predicting insulin resistance in African Americans [16]. On the other hand, a cross-sectional study of 6,546 Korean adults who underwent routine health examinations found that TG/HDL-C was a consistent indicator of insulin resistance in subjects without metabolic syndrome [17].

In addition, depending on ethnicity, different thresholds of TG/HDL-C have been proposed for predicting insulin resistance. A ratio of 3.0 was suggested for non-Hispanic whites and Mexican Americans, 2.0 for non-Hispanic blacks [18], and 2.5 for African-American men [19]. Despite of numerous studies have evaluated the optimal threshold for TG/HDL-C, to the best of our knowledge, no studies have specifically compared the accuracy of TG/HDL-C with and without other clinical and demographic factors in predicting insulin resistance. Moreover, no studies have reported that prediction of insulin resistance using TG/HDL-C in Taiwanese adults. Hence, the aim of this study was to evaluate the prediction of insulin resistance by TG/HDL-C with and without the inclusion of other clinical available factors for clinical applications.

## Methods

### Study Subjects

Participants were recruited at the time of their general health examination at the Buddhist Dalin Tzu Chi General Hospital located in Chiayi county, Taiwan between May 2007 and April 2008. The study hospital is located in south Taiwan and most of the participants came from Yulin, Chiayi, Tainan, and Kaoshiung counties in southern Taiwan.

This study was approved by the Institutional Review Board of the study hospital and written informed consent was given by all the participants before enrollment.

Information on demographic and clinical characteristics including age, sex, location of residence, weight, height, waist circumference, and sitting blood pressure were collected. Body mass index was calculated by dividing weight (in kilograms) by the square of the height (in meters). Daily energy expenditure was estimated using a seven-day physical activity recall questionnaire (Chinese version) [20].

### Sample processing and analyses

Triglyceride, total cholesterol, HDL-C, LDL-C, glucose, insulin, and alaninne aminotrasferase (ALT) were measured using blood sample collected from each subject after a minimum eight hour fasting period. Plasma fasting insulin concentrations were measured using human insulin Enzyme Linked Immunosorbent Assay (ELISA) kit (BioSource Europe S.A., Nivelles, Belgium). Insulin resistance was defined by homeostasis model assessment for insulin resistance (HOMA-IR). HOMA-IR was calculated by dividing the product of fasting plasma glucose (mg/dL) and fasting plasma insulin (mU/L) by 405 [5]. Other biochemical variables including blood lipids, and glucose were analyzed using an auto-analyzer (Sysmex XE-2100 Blood Cell Analyzer, Kobe, Japan).

### Statistical Analyses

Statistical analysis was performed using the *R*, version 2.12.1, software (Free Software Foundation, Inc., Boston, MA, U.S.A.). Subjects were divided into elevated HOMA-IR and low HOMA-IR groups using a cutoff value of 2. Two-sided *p *value ≤ 0.05 was considered statistically significant. Continuous data were expressed as mean ± standard deviation (SD) and categorical variables were represented by frequency and percentage. Differences in demographic and clinical characteristics of subjects between the low and elevated HOMA-IR groups were examined using the two-sample *t *test for continuous variables and Fisher's exact test for categorical variables.

Simple logistic regression was used to obtain probability of insulin resistance (HOMA-IR > 2) using only TG/HDL-C (Model 1). Multiple logistic regression analysis with stepwise variable selection procedure using the Akaike's Information Criterion (AIC) was also conducted to evaluate other factors that were significantly associated with insulin resistance in addition to TG/HDL-C (Model 2). A ROC analysis was conducted to evaluate the ability of the two models for correctly discriminating the subjects of low and elevated HOMA-IR. Plots of the sensitivity (true positive) versus 1-specificity (false positive) for the two models were made and the overall diagnostic accuracy between the two models was quantified using AUC. These two correlated ROC curves were then compared by applying the ROC test of DeLong using the roc.test function of the *pROC *library in *R*.

## Results

A total of 812 subjects were recruited into the study after excluding 8 subjects using lipid-lowering medications, 53 subjects using antidiabetic medications, and 4 using both types of medications. Table 1 shows the demographic and clinical characteristics of subjects categorized by HOMA-IR using a cutoff value of 2. The subjects comprised of 403 men aged 52.0 ± 11.0 [range 22.0-80.5] years and 409 women aged 52.1 ± 9.8 [range 19.3-81.5]. There were no significant differences in age, sex, location of residence, body height, total cholesterol levels, low-density lipoprotein-cholesterol (LDL-C) levels, and daily energy expenditure between the two HOMA-IR groups. The mean body weight, body mass index, waist circumference, systolic blood pressure, diastolic blood pressure, fasting glucose level, triglyceride level, alanine transaminase (ALT) level, and insulin level were significantly higher in the elevated HOMA-IR group. The mean high density lipoprotein-cholesterol (HDL-C) level was significantly lower in the elevated HOMA-IR group.

**Table 1:** Demographic and clinical characteristics of subjects categorized by HOMA-IR (N = 812)

Variable	HOMA-IR ≤ 2 n = 593	HOMA-IR > 2 n = 219	*P*
Age (years)	51.8 ± 10.3	52.9 ± 10.6	0.177
Sex			
Male	290 (48.9)^†^	113 (51.6)	0.496
Female	303 (51.1)	106 (48.4)	
Location of residence			
Metropolitan	240 (40.5)	90 (41.3)^‡^	0.835
Other	353 (59.5)	128 (58.7)	
Body weight (kg)	61.6 ± 9.99	66.4 ± 12.4	< 0.001
Body height (cm)	161.9 ± 8.1	161.8 ± 8.0	0.799
Body mass index (kg/m^2^)	23.4 ± 2.9	25.3 ± 3.8	< 0.001
Waist circumference (cm)	76.9 ± 8.3	82.5 ± 10.0	< 0.001
Systolic blood pressure (mmHg)	125.3 ± 18.8	129.0 ± 19.8	0.014
Diastolic blood pressure (mmHg)	73.6 ± 12.1	76.6 ± 12.0	0.002
Fasting glucose (mg/dL)	88.5 ± 10.0	93.6 ± 11.7	< 0.001
Triglyceride (mg/dL)	102.9 ± 59.5	137.1 ± 79.9	< 0.001
Total cholesterol (mg/dL)	192.7 ± 34.4	192.6 ± 38.2	0.968
HDL-C (mg/dL)	56.3 ± 14.8	50.5 ± 14.3	< 0.001
LDL-C (mg/dL)	127.5 ± 31.6	128.4 ± 35.3	0.738
Insulin (mU/L)	4.84 ± 2.06	15.66 ± 10.32	< 0.001
ALT (mg/dL)	24.2 ± 15.8	38.1 ± 50.2	< 0.001
Energy expenditure (Kcal×10^3^/day)	2.96 ± 1.43	3.03 ± 1.12	0.517

* Continuous variables were represented by mean ± standard deviation.

† Categorical variables were expressed as number (percentage).

^‡ ^One missing data for HOMA-IR > 2 in location of residence.

HDL-C: high-density lipoprotein-cholesterol

LDL-C: low-density lipoprotein-cholesterol

ALT: alanine transaminase

**Figure 1:**
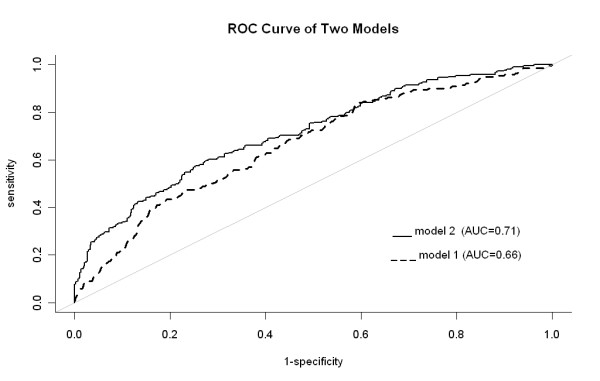
**The receiver operating characteristics curve between the two models**. Model 1 was obtained from the logistic regression of triglyceride-to-high-density lipoprotein cholesterol ratio (TG/HDL-C) with the area under the curve = 0.66; Model 2 was obtained from the multiple logistic regression with the area under the curve = 0.71. The *P*-value of the differences between the two areas was 0.007.

Table 2 shows the two models of the association between TG/HDL-C and HOMA-IR. Model 1 contained only TG/HDL-C as a predictor and Model 2 contained covariates in addition to TG/HDL-C that were obtained from stepwise multiple logistic regression. Female sex, greater waist circumferences, higher ALT levels, and higher TG/HDL-C were significantly associated with the prevalence odds of elevated HOMA-IR. In Model 1, an increase of 1 unit in TG/HDL-C ratio was associated with an elevation in the insulin resistance by 31% while in Model 2, the corresponding in the TG/HDL ratio was associated with an 19% elevation in the odds. Figure 1 shows the ROC curves between the two models. The AUC of Model 2 was 0.71 [95% CI = 0.67-0.75], which was significantly higher (*P *= 0.007) than the AUC 0.66 [95% CI = 0.62-0.71] of Model 1. The codes for calculating the probability of elevated insulin resistance using Model 2 in the R environment, Microsoft Excel, and OpenOffice Calc are provided in Appendix 1. Note that the model should only be applied to ranges within the observed values, i.e., waist circumference between 57.1 cm and 111.8 cm, ALT between 8 mg/dL and 526 mg/dL, and TG/HDL-C ratio between 0.26 and 22.26.

**Table 2:** Two logistic regression models for predicting insulin resistance (HOMA-IR > 2)

Variable	β	Standard error	*P*	Odds ratio	95% confidence interval
**Model 1**					

Intercept	-1.642	0.135	< 0.001		
TG/HDL-C	0.257	0.042	< 0.001	1.29	1.19-1.41

**Model 2**					

Intercept	-7.941	0.986	< 0.001		
Sex					
Male	1				
Female	0.933	0.207	< 0.001	2.54	1.70-3.84
Waist circumference (cm)	0.071	0.012	< 0.001	1.07	1.05-1.10
ALT (mg/dL)	0.015	0.004	< 0.001	1.01	1.01-1.02
TG/HDL-C	0.173	0.044	< 0.001	1.19	1.09-1.30

TG/HDL-C: triglyceride to high-density lipoprotein cholesterol ratio

ALT: alanine transaminase

## Discussion

This study aimed to develop a simple predictive model as a clinical tool for evaluation of insulin resistance. In clinical settings, it would be useful to identify individuals with insulin resistance using only routinely collected blood test results such as triglyceride and high-density lipoprotein cholesterol. Hence, we proposed the prediction of insulin resistance by the ratio of TG and HDL-C.

One unique finding in this study was that we evaluated the accuracy of prediction of insulin resistance using TG/HDL-C with or without the presence of other readily available demographic and biochemical variables. The model that included three factors in addition to TG/HDL-C significantly increased the predicting probability of higher insulin resistance compared with the model containing only TG/HDL-C. The three additional factors were sex, waist circumferences, and ALT levels, which are readily available measurements in clinical settings.

Previous studies had used different cutoff values of TG/HDL-C to predict insulin resistance. However, the results were inconclusive. The cutoff point for TG/HDL-C appeared to vary among different ethnic groups. It was reported to be 3.0 for non-Hispanic whites and Mexican Americans and 2.0 for non-Hispanic blacks [18]. In contrast to these studies, the present study did not use a cutoff point for TG/HDL-C to predict insulin resistance. Instead, we treated TG/HDL-C as a continuous variable to predict the probability of insulin resistance as measured by HOMA-IR. In a study of 1,824 Iranian men of age 40 years and above, it was reported that the prevalence of metabolic syndrome in subjects with TG/HDL-C greater or equal to 6.9 was 63.6%, compared with 3.0% in those with TG/HDL-C below 2.8 [21]. The risk of metabolic syndrome appeared to be incrementally associated with TG/HDL-C and there are no consensual cutoff values for it. Therefore, no cutoff point was set for TG/HDL-C in the present study.

In addition to the potential use as a predictor of insulin resistance in clinical settings, the ratio of TG and HDL-C has also been reported as a strong predictor of incident hypertension in a prospective study of 2831 non-hypertensive Middle Eastern women [22]. In addition, based on the findings from a study of 585 males with type 2 diabetes mellitus, log(TG)/HDL-C was suggested to be used as a simple means of estimating atherogenic dyslipidemia, which is closely associated with the future risk of coronary artery disease [23]. Moreover, in a prospective study of a nationally representative sample of United States adults with diagnosed diabetes, higher serum non-HDL-c concentrations was found to be associated with increased risk of death from cardiovascular diseases [24]. In our study, higher serum non-HDL-c levels were directly and significantly correlated with levels of TG/HDL-C, with a Pearson correlation coefficient of 0.34.

There are potential limitations regarding the interpretation of our results. First, previous research found that ethnicity affects the associations between TG/HDL-C and insulin resistance. The accuracy of the obtained model when apply to other ethnic groups will need additional evaluation. Second, the applicability of the model is limited to the observed data range of the present study.

## Conclusions

Insulin resistance is the core of the metabolic syndrome and a pre-diabetes condition. The present study demonstrated that the diagnostic accuracy of insulin resistance, defined by HOMA-IR, using TG/HDL-C can be significantly enhanced in the prediction model by including sex, waist circumference, and ALT, which are readily available clinical measurements. The model described in this report could be served as a tool to assist health care professionals to easily and accurately predict insulin resistance and thereby providing timely lifestyle modifications advice to susceptible individuals.

## Abbreviations

HOMA-IR: homeostasis model assessment for insulin resistance; TG/HDL-C: triglyceride to high-density lipoprotein cholesterol ratio

## Competing interests

The authors declare that they have no competing interests.

## Authors' contributions

JKC (CHIANG) analyzed the data and wrote the manuscript. NSL contributed to the discussion. JKC (CHANG) wrote the manuscript. MK edited the manuscript and contributed to the discussion. All authors read and approved the final manuscript.

## Appendix 1

Programming code in R, Microsoft Excel and OpenOffice Calc for calculating the probability of elevated insulin resistance (HOMA-IR > 2) based on the multiple logistic regression model.

### 1. In R environment

To calculate the probability of insulin resistance (HOMA-IR > 2), substitute the values for the variables X_1 _to X_4 _in the following regression equation:

yhat < - (-7.941                     # constant

+ 0.933*X_1 _                          #X_1 _= sex (female = 1, male = 0)

+ 0.071*X_2 _                          #X_2 _= waist circumference (cm)

+ 0.015*X_3 _                          #X_3 _= ALT (mg/dL)

+ 0.173*X_4 _                          #X_4 _= TG/HDL-C

)

phat < - 1/(exp(-(yhat))+1)

phat                                      # copy these syntax and paste on the R console

### 2. In Microsoft Excel or OpenOffice Calc

Key in the values for sex (female = 1, male = 0) in the A1 cell, waist circumference (cm) in the A2 cell, ALT (mg/dL) in the A3 cell, and TG/HDL-C in the A4 cell.

Key in the following formula in any empty cell on the spreadsheet to obtain the probability of insulin resistance.

= 1∕EXP(-(-7.941+0.933*A1+0.071*A2+0.015*A3+0.173*A4)+1)

Note: The model should only be applied to individuals with waist circumference between 57.1 cm and 111.8 cm, ALT between 8 mg/dL and 526 mg/dL, and TG/HDL-C between 0.26 and 22.26.
